# A Review Focusing on Microbial Vertical Transmission during Sow Pregnancy

**DOI:** 10.3390/vetsci10020123

**Published:** 2023-02-06

**Authors:** Shengjun Liu, Zixi Zhang, Longteng Ma

**Affiliations:** 1 Jiangxi Haida Feed Co., Ltd., Nanchang 331700, China; 2 Animal Nutritional Genome and Germplasm Innovation Research Center, College of Animal Science and Technology, Hunan Agricultural University, Changsha 410128, China

**Keywords:** microorganisms, vertical transmission, placental microbiome, maternal microbiome, breast milk microbiome

## Abstract

**Simple Summary:**

Neonates are highly susceptible to intestinal infections; this has been generally ascribed to the immaturity of the immune system, but other factors might contribute. The composition of the gut microbiota is a key factor, as the microbiota protects the host against colonization by pathogens. The neonatal gut microbiota is less diverse and lacks two taxa that are dominant in older intestines: members of the orders Clostridiales and Bacteroidales. Microorganisms are closely related to the body’s physiological activities and growth and development of the body, and participate in many physiological metabolic activities. Analysis of the structure and source of early colonizing bacteria in the intestinal tract of humans and rodents shows that early colonizing bacteria in the intestinal tract of mammals have solid maternal characteristics, and maternal microbes play an essential role in the formation of progeny intestinal flora.

**Abstract:**

Microorganisms are closely related to the body’s physiological activities and growth and development of the body, and participate in many physiological metabolic activities. Analysis of the structure and source of early colonizing bacteria in the intestinal tract of humans and rodents shows that early colonizing bacteria in the intestinal tract of mammals have solid maternal characteristics, and maternal microbes play an essential role in the formation of progeny intestinal flora. The placental microbiome, maternal microbiome and breast milk microbiome are currently hot topics in the field of life science. This paper discusses the vertical transmission and endogenous sources of the mother-to-piglet microbiome through these three pathways, aiming to provide a new research idea for intervention in the intestinal microbiome in young piglets.

## 1. Introduction

In large-scale pig production, sows and piglets are the key links to determine the production level and economic benefits of pig farms. At the same time, the corresponding gestation period, lactation period and newborn period are the core stages of feeding management of sows and piglets in large-scale pig production.

Intestinal microorganisms are closely related to the body’s physiological activities and growth and development of the body, and participate in many physiological metabolic activities. Existing studies have described the microorganisms in animals as independent physiological organs [1]. Intestinal health is a crucial factor affecting piglets’ performance and survival rate. Therefore, improving the intestinal health of piglets and avoiding or reducing intestinal pathogenic microbial infection are important issues to be solved in pig production [2]. Gut microbes do not just have beneficial or detrimental effects on their hosts. On the one hand, native intestinal microorganisms play a vital role in the development and maturation of the intestinal immune system and compete with harmful bacteria for colonization sites to avoid colonization of harmful bacteria [3]. On the other hand, intestinal microorganisms compete with the host for nutrients, fermenting undigested nutrients such as fibers, producing bacterial proteins or synthesizing them into vitamins, etc., for use in the host [4]. Factors such as the underdeveloped intestinal immune system of newborn piglets, the unestablished intestinal microecological environment balance and the susceptibility of weaned piglets to pathogenic microorganisms due to weaning stress are the main factors leading to growth stagnation and disease occurrence in piglets [5]. Recent studies have shown that the establishment and balance of the intestinal microflora structure can promote the development and maturation of the intestinal immune system in piglets, improve the intestinal function of newborn and weaned piglets and play an essential role in the establishment and function of normal intestinal homeostasis in piglets [6].

Although the importance of intestinal flora and the rule of colonization have been revealed, the origin of early intestinal flora and the earliest time of colonization have not yet been determined. The traditional view is that the intestinal tract in piglets is in a sterile state before delivery, and microbial colonization begins gradually through contact with food, the external environment, the maternal birth canal and feces, then evolves [7]. However, analysis of the structure and source of early colonizing bacteria in the intestinal tract of humans and rodents shows that early colonizing bacteria in the intestinal tract of mammals have solid maternal characteristics, and maternal microbes play an essential role in the formation of progeny intestinal flora [8]. After birth, piglets must come into contact with the sows’ skin, feces, urine and dwelling environment, and the microorganisms from these sites may enter the piglets’ intestines, but this is not discussed in this paper [7]. The placental microbiome, maternal microbiome and breast milk microbiome are currently hot topics in the field of life science. This paper discusses the vertical transmission and endogenous sources of the mother-to-calf microbiome through these three pathways, aiming to provide a new research idea for intervention in the intestinal microbiome in young piglets.

## 2. The Physiological Basis of Vertical Transmission

Intestinal microorganisms are involved in many physiological and metabolic processes in the host and may be involved in the maternal pregnancy process. Previous studies have shown that maternal gut microbes undergo drastic changes during pregnancy. Compared with early pregnancy, the intestinal flora in late pregnancy can promote host fat deposition and improve insulin tolerance, suggesting that intestinal microbes are involved in metabolic changes during maternal pregnancy [9]. In addition, intestinal microbes during pregnancy also play a role in the development and maturation of the offspring’s immune system. Short-chain fatty acids (SCFAs) are a significant fermentation metabolite of intestinal microorganisms. Other studies have shown that short-chain fatty acids produced by the fermentation of maternal intestinal microorganisms can also be transmitted to the offspring and promote the maturation and development of the offspring’s immune system [10,11]. During lactation, maternal intestinal microorganisms can be transferred to the offspring through breast milk through vertical transmission and affect the development of the offspring’s immune system [12]. In conclusion, it is of great importance to study the structure of maternal intestinal flora and the changes in its metabolites during the reproductive cycle, and to understand the role of intestinal microorganisms in the metabolic and immune changes of the mother during the pregnancy–lactation period and the healthy development of the offspring.

Many microorganisms colonize the intestinal tract of mammals, which profoundly impact on host nutrient metabolism and immune function [13]. The intestinal flora is involved in the activation of the host immune system and plays an essential role in constructing the intestinal barrier function [14]. The colonization of intestinal flora after birth is affected by the oxygen environment in the intestinal cavity: with oxygen consumption, microorganisms colonize the intestinal tract in the order of aerobic, facultative and obligate anaerobic, and eventually form microbial flora changes of different types and compositions from the anterior intestinal segment to the posterior intestinal segment [15]. The neonatal intestinal colonizing bacteria in mammals are dominated by facultative anaerobes such as Streptococcus and Staphylococcus. After that, they are quickly replaced by Bifidobacterium, Corynebacterium, Lactobacillus, Ruminococcus [15]. After birth, piglets quickly build up breast-dependent intestinal flora. Later, due to intestinal environment changes brought about by gastrointestinal tract development, gradually enriched dietary structure, weaning stress and other factors, the colonization sites of specific intestinal flora are transformed, and the microbial diversity is gradually enriched [16].

Gram-negative bacteria infections include the following: Brucellosis [17], Escherichia coli enteritis [18,19] and Salmonellosis [20]. Gram-negative bacteria are encased in protective capsules. The capsule prevents white blood cells (which fight infections) from engulfing the bacteria. Gram-negative bacteria have an outer layer beneath the capsule that helps them resist certain antibiotics (such as penicillin). The rupture of the outer membrane can release a toxin called endotoxin. Endotoxin is the main cause of severe symptoms after infection with Gram-negative bacteria. Although Gram-negative bacteria has been traditionally considered a pathogen mainly transmitted by direct contact, through the introduction of sub clinically infected animals into a previously uninfected herd, recent findings position Gram-negative bacteria as a potential threat for indirect transmission between farms [21].

Vertical transmission refers to the transmission of genetic material and microorganisms, such as bacteria and viruses, from parents to their offspring. Vertical transmission of microorganisms between the mother and child is widespread in mammals [22]. The migration of microorganisms in late pregnancy and lactation is an essential physiological basis for vertical transmission. Its internal mechanism stems from the combined action of the maternal immune function and hormone level. During pregnancy, material exchange between female piglets is mediated by the placenta, umbilical cord and amniotic fluid. During the preparation stage of childbirth, the interaction between microorganisms and the host immune–endocrine system makes the specific flora in the maternal gut and birth canal and all symbiotic flora in the mammary gland show progressive proliferation, which makes necessary preparation for the vertical transmission of beneficial flora to the offspring [23]. At the same time, there is apparent microbial migration between different parts of the mother, and microorganisms from different parts may migrate to the birth canal and mammary gland of sows through endogenous pathways. Blood flow provides the natural driving force for microbial migration, and monocytes and dendritic cells play an important role [24,25]. It has been proven that intestinal flora can break through the mucosal barrier in three ways, including penetrating tight junction proteins, invading intestinal epithelial cells and actively seizing dendritic cells [26]. The bacteria entering the blood can coexist with the immune system through some unknown mechanism, then enter the mammary gland along with the blood flow. The potential symbiotic mechanism may be the physiological basis of maternal microbiome migration through the endogenous pathway, which needs further study [27]. In addition, the microorganisms and their DNA imprinting that enter the intestinal tract at the early stage of an animal’s life may activate the construction and maturation of the host immune system, affect the colonization of subsequent microorganisms and enable the host to later develop tolerance (or susceptibility) to the occurrence of diseases [28]. Some scholars believe that there may be a short “window period” in the early stage of life, during which immune cells in the intestinal tract of animals may preferentially develop tolerance in response to antigenic stimuli so that bacteria are expected to colonize the intestinal mucosa and establish a symbiotic relationship with the host. It also has a continuous influence on the morphology of the intestinal epithelium, intestinal immune function and immune system development [29].

## 3. Maternal Transmission of Intestinal Microflora in Piglets

### 3.1. Vertical Transmission of Placenta

The traditional view is that the uterus and animal embryos are sterile, and the intestinal flora of piglets is gradually formed through contact with the mother, the environment and the diet during and after birth. With the deepening of research, microorganisms have been found in cord blood, amniotic fluid and meconium of newborn babies in a healthy pregnancy, which proves that a healthy uterus is not a sterile environment [30,31]. The most significant number of non-pathogenic Escherichia coli from the intestine has been identified in the placenta, suggesting that the intestine may also be a critical source of placental microorganisms [32]. The embryonic form of fetal intestinal flora may start to be established in the mother’s womb, and the traditional “blood” inheritance between the mother and her offspring may be accompanied by “mycelial vein” transmission through “placenta-umbilical blood” or “placenta-amniotic fluid” [33].

Some scholars studied the intestinal flora in the first, second and third trimesters of pregnancy and found that the maternal intestinal flora changed dramatically during pregnancy. The changes were similar to the intestinal flora structure of non-pregnant women suffering from metabolic syndrome. However, the intestinal flora structure associated with this “metabolic disease” was conducive to the development of pregnancy during pregnancy. It promotes the adaptive changes and regulation of maternal metabolism and immunity during pregnancy [9]. In addition, intestinal microorganisms during maternal pregnancy can also enter the womb through vertical transmission routes, which may affect the course of the pregnancy and the immune development of the offspring [34]. Most importantly, the intestinal bacteria of germ-free female mice are colonized for a short period time during pregnancy, and the microbial molecules and metabolites produced by them can be transferred to the offspring through the placenta and breast milk, affecting the development of the offspring’s innate immune system [34]. During pregnancy, the mother undergoes drastic changes in physiological metabolism and immunity to adapt to and nourish the implantation and development of the embryo and ensure the successful completion of pregnancy [35]. In the early gestation period, the mother is in a state of anabolic metabolism. With the growth of the fetus, the mother’s metabolism is enhanced, manifested as increased appetite, digestive ability and weight gain, and she stores a large number of nutrients for the nutritional needs of the fetus in the late gestation period and lactation period. In addition, the ability of intestinal fat absorption during pregnancy is enhanced, the level of lipid and triglyceride is increased and more fat can be accumulated. However, due to the rapid growth and development of the fetus at the later stage of pregnancy, the nutrients absorbed by the mother are not enough to meet the nutritional needs of the fetus, and the nutrients stored by the mother in the early stage of pregnancy need to be consumed. The mother also changes from an anabolic state in the early stage of pregnancy to a relatively vigorous catabolic state, which is reflected in increased insulin tolerance and the consumption of maternal fat for energy supply. In terms of immunity, maternal inflammation is associated with the first trimester, which is conducive to blastocyst implantation and placenta formation. In contrast, during the second trimester, the fetus rapidly grows and develops, and the mother turns into an inflammatory state dominated by Th2 cells. An infection or inflammatory reaction at this stage is likely to induce abortion or premature birth of the fetus. In the late gestation period, the fetus completes its growth and development, and the mother becomes an inflammatory state again. The levels of tumor necrosis factor (TNF-a) and interleukin 6 (IL-6) in the blood are significantly increased, among which the pro-inflammatory factor NF-KB plays an important role in initiating labor. Similarly, the hormones and metabolism of the lactating sows also undergo complex changes to initiate and maintain lactation. Compared to the gestation period, the mother has a higher level of nutritional requirements during lactation. In addition to dietary energy, the mother stops fat deposition and mobilizes the fat and energy stored during pregnancy to be transferred to the mammary gland for milk synthesis. In addition, the maternal blood sugar in lactation also gradually recovers from the high level of pregnancy to an average level, and insulin resistance also gradually decreases. The metabolic and immune changes in sows during gestation and lactation are conducive to fetal development and growth. The destruction of these adaptive changes may lead to premature delivery and even abortion. Statistics show that in commercial genetic lines, the prenatal loss of pig litter size ranges from 30% to 50% [36]. Therefore, the healthy development of offspring needs to ensure the normal metabolic and immune changes during the maternal reproductive cycle.

The placenta is a unique organ that connects the mother and protects the fetus in utero during pregnancy. It is comprised of tissues such as an amniotic membrane, dense chorionic membrane and decidua basalis [37]. The placenta establishes an immune interface between the mother and fetus that can maintain the pregnancy status and reduce the rejection of the fetus by the maternal immune system. It also serves as a transport platform for nutritional and metabolic waste between the mother and fetus and can produce various peptides and steroids to regulate the related metabolism and development of the mother and fetus [38]. The traditional view is that the placenta is sterile. Satokari identified Lactobacillus and Bifidobacterium DNA in the human placenta for the first time [39]. Later, Aagaard studied placenta slices obtained aseptically. High-throughput sequencing was used to analyze the existence of Firmicutes, Tenericutes, Proteobacteria, Bacteroidetes, Fusobacteria and other bacteria. The presence of bacteria in different areas of the placenta was observed and recorded by morphology. The results also confirmed that a low abundance but metabolically rich microbiome inhabited the placenta [32]. These results suggest that there may be placental and amniotic fluid-mediated microbiome transmission between the mother and fetus prior to delivery. Further studies suggest that the colonization of intestinal flora may begin during the embryonic gastrointestinal tract formation stage. In a study on the intestinal flora structure and metabolites in Landrace sows and Rongchang sows during the reproductive cycle, it was found that the relative abundance of Actinobacteria and Coriobacteriaceae increased gradually with the change of gestation period. On the other hand, the abundance of Halomonas from Proteobacteria decreased gradually [40].

A blood or tissue DNA extraction kit was used to extract the DNA from the gastrointestinal tract and placenta tissue of Kunming pregnant mice at 12 to 13 days of pregnancy and 19 to 20 days of pregnancy, and high-throughput sequencing was conducted. The results showed that there were four dominant bacteria at the phylum level, Proteobacteria (95.00%, 88.14%, 87.26%), Actinobacteria (1.16%, 4.10%, 3.38%), Bacteroidetes (1.71%, 2.15%, 2.63%) and Firmicutila (0.75%, 2.62%, 2.01%). There were nine dominant bacteria families at the family level. Among them, Enterobacteriaceae (46.99%, 44.34%, 41.08%), Shivaraceae (21.99%, 21.10%, 19.05%) and Moraxella (9.18%, 7.09%, 5.64%) were the leading families. However, the presence of microorganisms in the placenta remains controversial, although the vertical transmission of microorganisms between the mother and offspring during pregnancy has been consistently demonstrated. Lauder could not isolate bacterial DNA in the human placenta using a near-sterile approach [41]. It is generally believed in medicine that placental microorganisms are highly correlated with preterm birth, but the exact colonization time of the intestinal flora before birth and its effect on the fetus still need further research [29].

### 3.2. Vertical Transmission of the Reproductive Tract

The birth canal is another important route for mother-to-child transmission. The intestinal flora structure in vaginal birth babies is similar to that of the mother’s birth canal microbes. Therefore, during the passage through the birth canal, the microbial symbiotic bacteria in the birth canal will be spread on the baby’s body surface. The microbial flora on the whole body of the baby is closely related to the way of delivery. While the babies delivered through C-section lack this process, their microbial colonization process is also affected [42,43].

During parturition, the microflora in the maternal birth canal may also be an important source of intestinal probiotics for piglets. The widely colonized organisms in the birth canal of mammals are called the birth canal microbiome. In human beings, the birth canal microbes have been considered as an important barrier involved in protecting the host from various bacteria, fungi and viruses. A healthy and stable birth canal microecology is very important for a smooth pregnancy and delivery and is also an important source of early intestinal flora for cisinatal infants [44]. Some scholars have pointed out that children born via C-section have an increased risk of immune disorders and a higher incidence of chronic diseases such as asthma, anaphylaxis and inflammatory bowel disease than cisnatal infants [45]. Liu compared the fecal microbial test results of C-section neonates and vaginal neonates within 2 to 4 days of birth and found differences in the intestinal flora structure between the two. Four genera including Staphylococcus, Clostridium, Enterobacter and Streptococcus were the most common intestinal bacteria at this stage. In contrast, Escherichia coli, Bacteroides and Bifidobacterium longum were the dominant bacteria in the intestinal flora of vaginally born neonates.

Interestingly, this defect in Caesarean section appears to be partially offset by artificial inoculation after birth by applying gauze with birth canal microorganisms [33]. The abundance of Bacteroides was significantly reduced in C-section infants compared to vaginal births [43], and the effect persisted at 6 weeks of age [46] and even up to 4 months after birth [47]. However, other studies have shown that this difference gradually disappears after 6 weeks of age [48]. This means that the delivery mode significantly impacts the intestinal flora structure of early neonatal colonization, as well as subsequent physical health [49]. Studies have shown that the birth canal in healthy women is inhabited by rich lactic acid bacteria, including Lactobacillus acidophilus and Lactobacillus plantarum, which can use glycogen in the birth canal epithelium to produce lactic acid, acetic acid and other organic acids. The pH of the local environment in the birth canal is maintained at 3.8–4.4, and antibacterial substances such as hydrogen peroxide can be produced. In addition, competitive space occupation and other effects antagonize the growth and reproduction of disease-resistant pathogens, ensuring the health and stability of the birth canal microflora [50].

Further studies found that there was no significant difference in the diversity and abundance of Lactobacillus in the birth canal during the early, middle and late trimesters of pregnancy. However, the number of Lactobacillus increased rapidly in the short term before delivery, which may be a necessary preparation for the acquisition of maternal beneficial bacteria from the offspring intestine during subsequent delivery [51]. However, in most cases, the influence of the maternal tract microbes on the health of the offspring may not be significant. Sakwinska compared the fecal flora in infants with different delivery methods and feeding patterns and their mothers. The results showed that the intestinal flora in most infants was very limited in similarity to that of their mothers, which meant that only a tiny number of birth tract flora had been effectively colonized in the offspring’s intestines [52].

Animal experiments have also shown that the birth mode affects the intestinal flora structure in newborn piglets, and that piglets born naturally have a higher Bacteroides-Prevotella abundance [53]. It has been found that it may be transmitted into piglets through breast milk or ectopic stool, suggesting that vertical microbial transmission between the mother and child impacts the intestinal microbial colonization in piglets [54]. Two probiotics, Lactobacillus acidophilus and Bifidobacterium lactis were fed to pregnant sows by Buddington et al. It was found that the two probiotics could be detected in the birth canal and feces of the piglets. It might be suggested that the two probiotics colonized the intestinal tract of the piglets through vertical transmission routes in the birth canal [55].

### 3.3. Vertical Transmission of Milk

Breast milk is a very complex biological fluid, considered the most natural and safe source of nutrition for infant growth. Breast milk (especially colostrum) is rich in proteins, lipids, sugars, immune cells and bioactive molecules. It not only meets the nutrition needed for the rapid growth of infants, but also has anti-inflammatory and anti-infection functions. Milk provides up to 30% of the intestinal microbes for infants. It plays an important role in the colonizing and developing of neonatal intestinal flora [56]. Microbial transfer in the skin and milk changes the immune response of infants from Th2-dominated in the womb before birth to a Th1/Th2-balanced immune response after birth [57]. The microorganisms in milk can also stimulate the production of anti-inflammatory cytokines in the infants’ intestines and stimulate an intestinal anti-inflammatory response. These microorganisms can also reduce the risk of inflammatory diseases, immune-mediated asthma, atopic dermatitis and other diseases, prevent respiratory diseases and diarrhea, and protect the healthy intestinal development of infants [58]. Therefore, the microbial composition in milk is essential for the colonization and development of infant intestinal flora and the maturation of the intestinal immune system.

A large number of studies have proved that healthy mammalian breast milk contains a high concentration of microbes, with a content as high as 10^4^–10^5^, including Lactobacillus plantarum [59], Bifidobacterium [60], Ruminococcus bromii, Coprococcus [60], and Escherichia coli [61], Streptococcus, Veillonella, Rothia and other microorganisms [56]. This result was consistent with other studies [62], supporting a correlation between nutritional components and specific microbiome components [45,63]. Significant differences in the relative abundances of the most predominant genera were most apparent during the first 5 days of lactation, which might be due to changes in the nutritional components in the colostrum and the relative stability of nutrients in transitional and mature milk. Similar patterns of change were also observed at the species level. Lactobacillus reuteri, Lactobacillus mucosae and Akkermansia muciniphila are potential probiotic bacteria [64, 65, 66]. The relative abundances of these probiotics were shown to increase with lactation time in sow milk significantly and the piglet gut [67], while the potentially pathogenic Staphylococcus epidermidis generally decreased in our samples [68]. These microorganisms enter the offspring’s gastrointestinal tract with milk for colonization. It is a continuous source of early colonizing bacteria in the offspring’s gut.

The results of high-throughput sequencing of the human milk microbiome showed that the microbial diversity in milk exceeded the traditional hypothesis. The diversity changed gradually with the increase in lactation days. At the phylum level, Proteobacteria (41%), Firmicutes (35%) and Bacteroides (17%) dominated human milk at week 1, accounting for more than 93%. Firmicutes in human milk at week 3 showed the highest relative abundance (50%), and there were always more than 200 species in human milk at the genus level [59]. Many studies have shown that there is always a “core” microflora dominated by Streptococcus and Staphylococcus in milk, which is not affected by geographical location, maternal status and analysis methods. However, maternal health status, gestational order, mode of delivery, diet, genetic background and geographical location can affect or potentially affect the microbial diversity in milk [34,69]. It has been confirmed that microbial symbiosis exists objectively in humans, rodents and pigs, and microorganisms are distributed in the udder skin, milk ducts, milk lobules and adipose tissue [70]. Kemper first identified the existence of bacteria in the breast tissue of sows and confirmed that there were significant differences in the structure of the bacteria in the breast of sows before and after, while the effect of postpartum dyslactation syndrome was not significant [71]. The isolation and identification of lactic acid bacteria in the colostrum of sows and the intestinal tract of newborn piglets showed that the two have a great degree of homology, which means that the probiotics from breast milk can effectively colonize the intestinal tract of newborn piglets [72]. Chen conducted 16S rRNA high-throughput sequencing of 160 milk samples selected from 20 sows at 8 different time points. The analysis showed that the microorganisms in pig milk varied with lactation days but remained generally stable in composition and structure. The composition and diversity of the microorganisms in pig milk showed significant variability in colostrum while stable in transition and mature milk. Firmicutes and Proteobacteria always dominate in pig milk. At the genus level, Corynebacterium and Streptococcus in porcine colostrum were significantly higher than those in transition and mature milk. Two unknown genera of Lactobacillus, Ruminococcaceae and Lachnospiraceae, and one unnamed Clostridiales, as dominant bacteria, showed high relative abundance in transition and mature milk [40].

## 4. The Intestinal Flora of Sows Was Targeted

Matrilineal transmission of the intestinal flora in piglets refers to the transmission of commensal bacteria from parents to offspring, including transmission through germ cells, transmission through the placenta during pregnancy, transmission through the canal during delivery, perinatal transmission and postpartum transmission through milk. In addition, vertical transmission plays an important role as the initial transmission and influence mode of bacteria in the intestinal tract of piglets.

**Figure 1:**
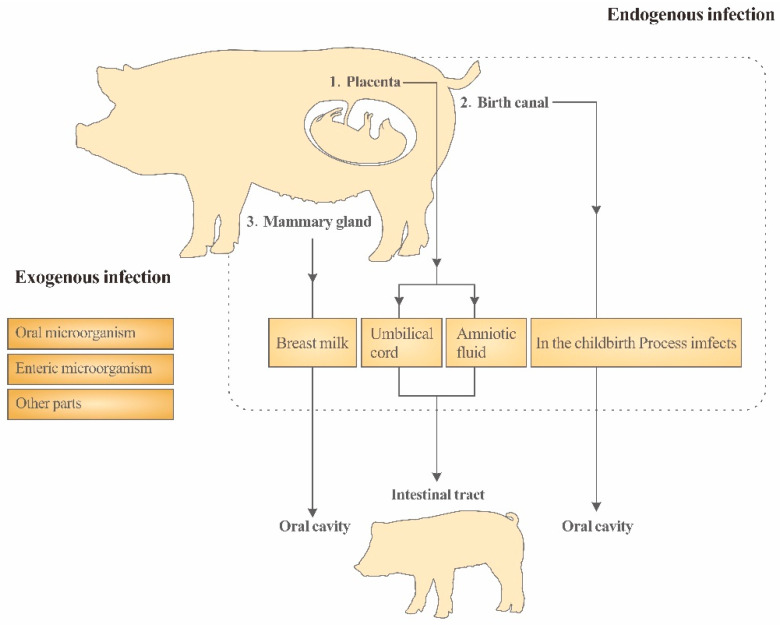
In the childbirth Process imfects to In the childbirth process infects.

Based on the microbial migration theory prevalent in mammals, the microbiome in every part of the sow’s body is likely to be transferred to the piglets [73]. However, considering the potential sources of the placenta, birth canal and breast milk microbes mentioned above, together with the possible influence of the sows’ feces on the intestinal flora of the piglets before weaning, the sows’ intestinal flora can enter the intestinal flora of the piglets through various ways and can continue to have the ability to mold the intestinal flora of the piglets over multiple periods so that it can be regarded as the most crucial source of the intestinal flora of the suckling piglets. The maternal transmission pathway of the intestinal flora in piglets is shown in Figure 1. Authorities have reported that within 4 months after delivery, the microbiome of the human mother’s skin, mouth, intestine, birth canal and other parts participate in shaping the progeny’s intestinal flora. Furthermore, with the continuous progress of lactation, the role of the maternal intestinal flora on the progeny’s intestinal flora gradually increases [74]. Therefore, it is worth attracting the attention of researchers to conduct a nutritional intervention on the intestinal flora structure of suckling piglets by targeting the intestinal flora of sows.

## 5. Conclusions

In conclusion, the maternal microbiome may colonize the intestinal tract of piglets through the placenta, birth canal and milk, so that the intestinal microflora of the offspring has a robust maternal origin. At the same time, the maternal microflora in the progeny intestinal tract has strong characteristics of the maternal intestinal flora, so that nutritional measures based on maternal intestinal microflora can also affect the intestinal microflora structure of the piglets. However, in the field of livestock production, there are still few reports on the vertical transmission of the microbiome. Therefore, the vertical transmission of the microbiome between female piglets, the related nutritional intervention and the changes in the structure of the microbiome are worthy of researchers’ attention. Further research on the vertical transmission of the microbiome will provide theoretical guidance for the development of the pig industry.

## Data Availability

Data openly available in a public repository.
